# Airway dimensional changes following bone anchored maxillary protraction: a systematic review

**DOI:** 10.1186/s12903-023-02940-0

**Published:** 2023-05-03

**Authors:** Samar M. Adel, Bassant A. Abbas, Wessam W. Marzouk, Abbas R. Zaher

**Affiliations:** 1 Lecturer, Department of Orthodontics, Faculty of Dentistry, Alexandria University, Champolion Street, Alexandria, El Azarita Egypt; 2 PhD resident, Department of Orthodontics, Faculty of Dentistry, Alexandria University, Alexandria, Egypt; 3 Professor, Department of Orthodontics, Faculty of Dentistry, Alexandria University, Alexandria, Egypt

**Keywords:** 3D airway changes, Class III, Bone-anchored, Maxillary protraction, CBCT

## Abstract

The introduction of skeletal anchorage utilized for maxillary protraction with a face mask or class III elastics has been developed for the management of class III malocclusions with minimal dental effect. The objective of the present review was to evaluate the current evidence regarding airway dimensional changes following bone-anchored maxillary protraction. A search was conducted by two authors (S.A & B.A) in the following databases: MEDLINE via PubMed, Cochrane Library, Web of Science, Scopus, Google Scholar and Open Grey; besides a hand search in references of selected articles and developing a search alert in electronic databases. Selection criteria comprised randomized as well as prospective clinical trials evaluating airway dimensional changes following bone-anchored maxillary protraction. Relevant data were extracted after studies retrieval and selection. The risk of bias was thereafter evaluated using the revised RoB 2 tool for randomized clinical trials and the ROBINS-I tool was used for non-randomized clinical trials. The quality of studies was assessed using the modified Jadad score. After examining (eligibility) full-text articles, four clinical trials were ultimately included. These studies evaluated the airway dimensional changes, following bone-anchored maxillary protraction in comparison to different control study groups. Based on the available evidence, all the bone-anchored maxillary protraction devices used in the eligible studies in the present systematic review resulted in an improvement in the airway dimensions. However, due to the few numbers of studies available and the guarded evidence due to the low quality of evidence of three out of four included articles, there is no strong evidence to support a significant increase in the airway dimensions following bone-anchored maxillary protraction. Therefore, there is a need for more randomized controlled clinical trials with similar bone-anchored protraction devices and similar assessment methods for more valid comparisons, excluding any confounding factors, on airway dimensional changes.

## Introduction

Class III malocclusion represents a growth-related dentofacial deformity with maxillomandibular problems in relation to each other and/or cranial base. Its prevalence greatly varies within different races, ethnic groups, and geographic regions. A multifactorial etiology has been proposed for class III malocclusions, which involves the expression of a moderate distortion of normal development as a result of interaction between genetic hereditary factors with environmental factors. Different skeletal topographies of underlying Class III malocclusion are due to discrepancy in the maxillary and mandibular growth compounded with vertical and/or transverse problems apart from sagittal malformations. About 30–40% of Class III malocclusions with skeletal origin is manifested by retrognathism or deficiency of the maxilla. The spectrum of complications for Class III malocclusion ranges from dentoalveolar problems with functional anterior shift of the mandible to true skeletal problems with serious maxillomandibular discrepancies, which makes its diagnosis and management highly challenging in growing children [1–5].

Concern regarding early treatment and the need for interceptive care in the case of Class III malocclusion has always been a dilemma, considering that not all problems can be addressed in these cases until maxillomandibular growth is further completed, and the long-term outcome of various treatment modalities may depend on the growth potential of an individual. However, interceptive treatment of Class III malocclusions should be undertaken if it prevents damage to the oral tissues, eliminates growth restrictions for more favorable growth and/or significantly reduces the amount or severity of future orthodontic and surgical interventions [1, 6, 7]. Previous literature has pointed out early interceptive treatment modalities for growing skeletal Class III malocclusions including extraoral, intraoral and skeletal anchorage systems either with extra oral [8] or intra oral [9, 10] traction. These skeletal anchorage devices include the use of skeletally anchored facemask appliance using two infrazygomatic miniplates, the use of four miniscrews or miniplates in the upper infrazygomatic and lower symphysis segments, and the use of bone anchored expanders with two lower symphysis miniplates [9, 10].

Going back in the literature, maxillary deficiency treatment at an early age was introduced primarily by the famous surgeon Delaire in 1976 [11] and the orthodontist Petit in 1983 using an extraoral facemask anchored on the dentition. Consequently, dentoalveolar effects were more pronounced than skeletal maxillary protraction, [12, 13] resulting in unwanted maxillary incisor protrusion, mandibular incisor retroclination, mesial movement and extrusion of maxillary molars dentally, as well as clockwise rotation of the mandible skeletally [14–16]. Skeletal anchorage applied in the maxillary buttress with a face mask has been first introduced to decrease dentoalveolar compensations [17–19]. Shortly after, De Clerck et al. [20] proposed the utilization of Class III elastics between skeletal mini plates anchored in the maxillary infrazygomatic crest posteriorly and mandibular symphysis anteriorly also termed bone anchored maxillary protraction or BAMP. Miniscrew or mini plate anchored maxillary protraction proved to be a promising treatment alternative for the management of growing skeletal Class III patients, avoiding all aforementioned side effects of tooth borne maxillary protraction [21].

Numerous studies evaluated the effects of maxillary protraction on pharyngeal airway dimensions and have revealed contradicting results [22–24]. Earlier studies using conventional 2D assessment showed that facemask therapy in combination with rapid maxillary expansion (RME) increased the pharyngeal airway dimensions [25–27]. On the contrary, others concluded that both the nasopharyngeal and oropharyngeal airway dimensions remained stable following maxillary protraction [28]. However, the 2-D cephalometric radiographs may hinder the precision of the linear and volumetric measurements of the upper airway [29–31]. Nowadays, cone-beam computed tomography (CBCT) has been extensively taken over to evaluate the upper airway shape and volume [22, 32–34]. CBCT presents the advantages of volumetric instead of merely linear measurements, and measurements without distortions that are not relying on head positioning [22, 33]. Chen and associates [22] utilized CBCT to assess changes in the upper airway dimensions following maxillary protraction using facemasks. They noticed a rise in the volume of the nasopharynx and oropharynx of developing patients with Class III malocclusion when compared to untreated Class III patients [22]. Additionally, using CBCT, Nguyen et al. [33] revealed an improvement in airway volume and oropharyngeal dimensions in patients treated with BAMP.

Recently, a meta-analysis of 6 studies demonstrated that maxillary protraction appliances can actually lengthen the nasopharynx and the posterior pharyngeal airways [26]. However, little was mentioned on the stability over the long term of the skeletal effect of the protracted maxilla or improvement in posterior airway dimensions.

Nowadays, there is no agreement in the literature regarding the exact airway changes after using bone-anchored maxillary protraction in the correction of maxillary deficiency in skeletally developing class III patients and its long-term stability. Therefore, the present systematic review was performed to appraise the available evidence in regard to airway dimensional changes after using different methods of bone anchor to protract the maxilla.

## Materials and methods

### Protocol and registration

**Fig. 1:**
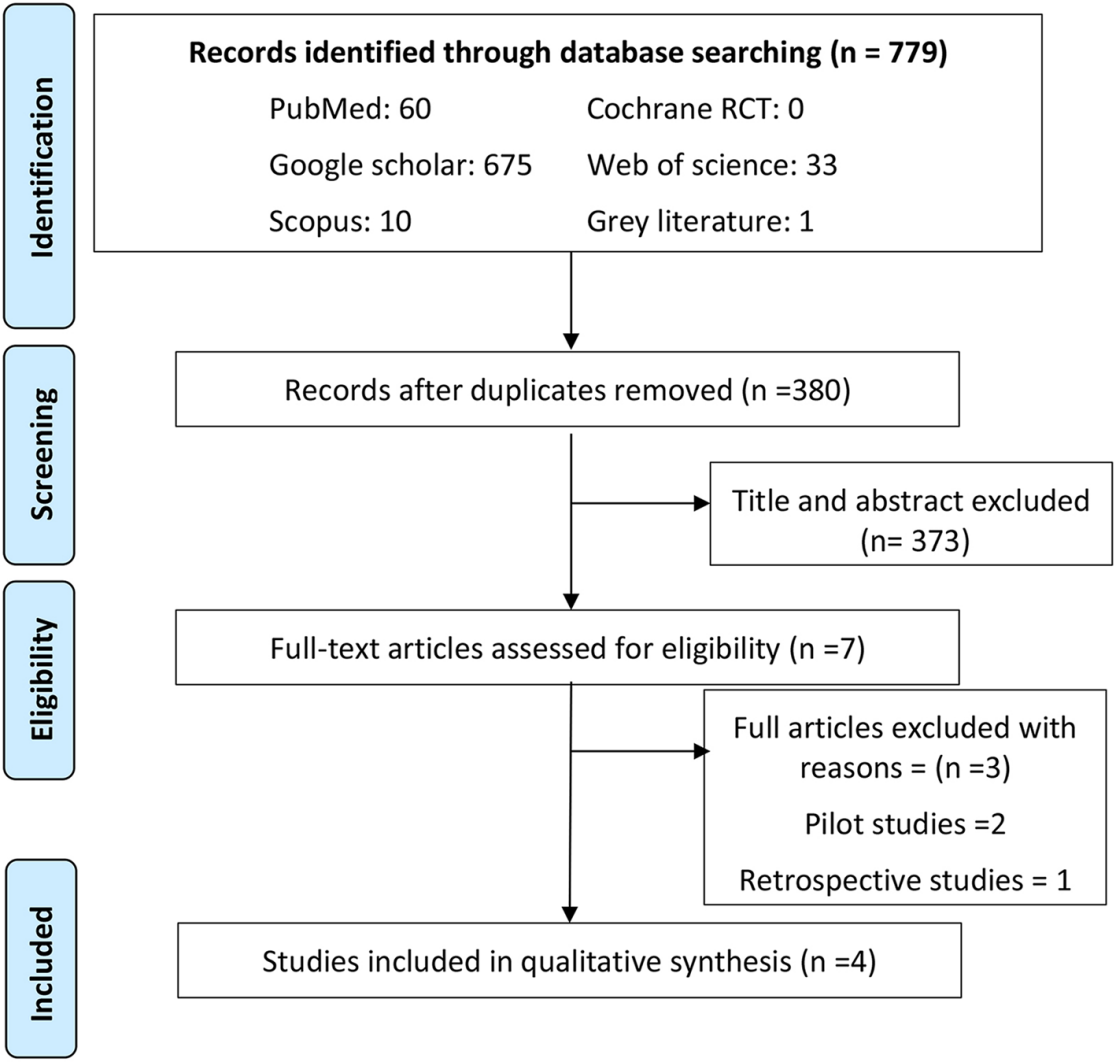
Flowchart of the study selection process based on PRISMA statement

The present review was conducted and reported following the Preferred Reporting Items for Systematic Reviews and Meta-Analyses (PRISMA) statement [35] (Fig. 1).

### Eligibility criteria

The current systematic review was conducted to tackle the following question: “Do airway dimensions change following bone-anchored maxillary protraction?

Based on PICOS [35] approach, the presented criteria were applied:

- Population (P): Human participants of any age or sex, having Class III malrelation with maxillary deficiency and/or obstructive sleep apnea.
- Intervention (I): Bone-anchored maxillary protraction
- Comparison (C): positive controls: using conventional expansion and tooth-borne face mask therapy, or negative controls: untreated group
- Outcome (O): Airway dimensions changes evaluated by 2D and 3D assessment methods
- Study design (S): Randomized clinical trials (RCTs) or non-randomized clinical trials (non-RCTs).

Literature reviews, systematic reviews, meta-analyses, patients with orthognathic surgeries, cleft lip and palate patients, patients with medical illnesses or presenting with craniofacial syndromes, animal studies, in vitro studies, retrospective studies, finite element analysis studies, case reports, pilot studies, conference papers, editorials and books were not eligible for inclusion.

### Information sources and search strategy

Studies were retrieved by searching the following electronic databases: MEDLINE via PubMed, Cochrane library, Web of Science and Scopus from the foundation of each database till the end of October 2022. No restrictions were applied in regard to the language or publication date during searching. Moreover, Google scholar and Open Grey were looked for grey literature. References of relevant articles were additionally searched and “Citation Networks” of relevant articles in Web of Science were checked to retrieve studies that could have been overlooked in the electronic database searches. An alert was created for each database using its relevant search strategy and monitored regularly to have a notice of any updated relevant study till the end of October 2022. The strategy for searching articles was first formulated for PubMed, then it was applied to the syntax rules of each database as presented in Table 1.

**Table 1:** Literature search conducted to identify studies (last search date October 30^th^, 2022)

Data base	Search	Search strategy	Hits
MEDLINE (via PubMed)	#1	(orthodontic anchorage procedures[MeSH Terms]) OR (miniscrew-supported[Title/Abstract])) OR (miniscrew assisted[Title/Abstract])) OR (miniscrew^a^[Title/Abstract])) OR (mini screw^a^[Title/Abstract])) OR (mini-implant^a^ [Title/Abstract])) OR (microimplant^a^ [Title/Abstract])) OR (micro-implant^a^ [Title/Abstract])) OR (bone borne[Title/Abstract])) OR (bone-anchor^a^ [Title/Abstract])) OR (skeletal anchorage[Title/Abstract])) OR (skeletally anchored[Title/Abstract]))) OR (mini plate^a^ [Title/Abstract])) OR (mini-plate^a^ [Title/Abstract])) OR (infrazygomatic screws^a^ [Title/Abstract])) OR (zygomatic plates^a^ [Title/Abstract])AND (Maxillary protraction[Title/Abstract])) OR (Maxillary protract^a^ [Title/Abstract]) Airway change^a^ [MeSH Terms] OR oropharyngeal change^a^ [Title/Abstract] OR airway dimensions [Title/Abstract] OR airway dimension^a^ [Title/Abstract] OR OSA [Title/Abstract] OR obstructive sleep apnea [Title/Abstract] AND Class III	369
	#2	[Title/Abstract] OR Class 3 [Title/Abstract] OR maxillary deficiency OR [Title/Abstract] maxillary retrusion [Title/Abstract] OR maxillary retrognathism [Title/Abstract])	1030
	#3	1 AND 2	**60**
Scopus	#1	TITLE-ABS-KEY ("mini screw-supported" OR "mini screw assisted" OR "miniscrew^a^" OR "mini screw^a^" OR "mini-implant^a^" OR "micro implant^a^" OR "micro-implant^a^" OR "bone borne" OR "bone-anchor^a^" OR "skeletal anchorage" OR "skeletally anchored" OR "mini plate^a^" OR "mini-plate^a^" OR "zygomatic plates^a^" AND "Maxillary protraction" OR "Maxillary protract^a^")	110
	#2	TITLE-ABS-KEY (“oropharyngeal change^a^” OR “airway dimensions” OR “airway dimension^a^” OR “OSA “OR “obstructive sleep apnea” AND “Class III” OR “Class 3” OR “maxillary deficiency” OR “maxillary retrusion” OR “maxillary retrognathism”)	213
	#3	1 AND 2	**10**
Cochrane	#1	[mh “orthodontic anchorage procedures”] OR miniscrew-supported:ti,ab,kw OR miniscrew-assisted:ti,ab,kw OR miniscrew:ti,ab,kw OR miniscrews:ti,ab,kw OR mini-screw:ti,ab,kw OR mini-screws:ti,ab,kw OR mini-implant:ti,ab,kw OR mini-implants:ti,ab,kw OR microimplant:ti,ab,kw OR microimplants:ti,ab,kw OR micro-implant:ti,ab,kw OR micro-implants:ti,ab,kw NEXT bone-borne:ti,ab,kw OR bone-anchored:ti,ab,kw NEXT skeletal anchorage:ti,ab,kw OR skeletally-anchored:ti,ab,kw NEXT mini plate^a^:ti,ab,kw OR mini-plate^a^:ti,ab,kw OR infrazygomatic screws^a^:ti,ab,kw OR zygomatic plates^a^:ti,ab,kw AND Maxillary protraction:ti,ab,kw OR Maxillary protract^a^:ti,ab,kw	403
	#2	[mh “Airway change^a^ “]OR oropharyngeal change^a^:ti,ab,kw OR airway dimension^a^:ti,ab,kw NEXT OSA:ti,ab,kw OR obstructive sleep apnea:ti,ab,kw AND Class III:ti,ab,kw OR Class 3:ti,ab,kw NEXT maxillary deficiency:ti,ab,kw OR maxillary retrusion:ti,ab,kw OR maxillary retrognathism:ti,ab,kw	0
	#3	1 AND 2	**0**
Web of science	#1	TI = (orthodontic anchorage procedures OR mini screw-supported OR mini screw assisted OR miniscrew^a^ OR mini screw^a^ OR mini-implant^a^ OR micro implant^a^ OR micro-implant^a^ OR bone borne OR bone-anchor^a^ OR skeletal anchorage OR skeletally anchored OR mini plate^a^ OR mini-plate*OR zygomatic plates^a^ AND Maxillary protraction OR Maxillary protract^a^)	3901
	#2	TI = (Airway change^a^ OR oropharyngeal change^a^ OR airway dimensions OR airway dimension^a^ OR OSA OR obstructive sleep apnea AND Class III OR Class 3 OR maxillary deficiency OR maxillary retrusion OR maxillary retrognathism)	10,827
	#3	1 AND 2	**33**
Google scholar https://scholar.google.com.eg/	#1	Allintitle:(orthodontic anchorage procedures OR mini screw-supported OR mini screw assisted OR miniscrew^a^ OR mini screw^a^ OR mini-implant^a^ OR micro implant^a^ OR micro-implant^a^ OR bone borne OR bone-anchor^a^ OR skeletal anchorage OR skeletally anchored OR mini plate^a^ OR mini-plate^a^OR zygomatic plates^a^ AND Maxillary protraction OR Maxillary protract^a^) AND	614
	#2	(Airway change^a^ OR oropharyngeal change^a^ OR airway dimensions OR airway dimension^a^ OR OSA OR obstructive sleep apnea AND Class III OR Class 3 OR maxillary deficiency OR maxillary retrusion OR maxillary retrognathism)	1680
	#3	1 AND 2	**675**
Grey literature http://www.opengrey.eu/	#1	(orthodontic anchorage procedures OR mini screw-supported OR mini screw assisted OR miniscrew^a^ OR mini screw^a^ OR mini-implant^a^ OR micro implant^a^ OR micro-implant^a^ OR bone borne OR bone-anchor^a^ OR skeletal anchorage OR skeletally anchored OR mini plate^a^ OR mini-plate^a^OR zygomatic plates^a^ AND Maxillary protraction OR Maxillary protract^a^)	220
	#2	(Airway change^a^ OR oropharyngeal change^a^ OR airway dimensions OR airway dimension^a^ OR OSA OR obstructive sleep apnea AND Class III OR Class 3 OR maxillary deficiency OR maxillary retrusion OR maxillary retrognathism)	1138
	#3	1 AND 2	**1**

^a,^^#^These values are part of the search strategies that were applied in the named databases. The bold values represent the final number of records that match the search in each database

### Study selection

All possibly relevant titles and abstracts were imported into a reference manager (EndNote X9, Thomson Reuters) and duplicates were deleted. Screening was independently accomplished by two reviewers (S.A, B.A), and articles were ranked based on data given by the title and abstract as “Excluded” or “Potentially eligible.” Articles were retrieved in full if they were scored by at least one of the reviewers as “Potentially eligible.” Assessment of eligibility was done on full- text articles by the same two reviewers and disagreements were resolved by discussion and consensus. If no agreement could be achieved, then a third reviewer was consulted (A.Z).

### Data collection

A data extraction sheet was formulated to extract the following information from eligible studies: author, publication year, journal, study design, study setting, details of participants (sample size, mean age, sex distribution), details of bone anchored maxillary protraction devices (location, type of bone anchorage, protraction protocol and the conjunctive use of expanders), assessment method, observation period, outcomes and results. The two reviewers extracted the data (S.A and B.A) and revised them. (Table 2).

**Table 2:** Characteristics of the included studies

Author; publication year; journal	Study design; study setting	Sample size(F/M); mean age	Location of anchor; protraction protocol ± expansion	Assessment method; observation period	Outcome measurements of airway	Results
Miranda et al., [10] 2021 Clin Oral Invest	Single centered randomized controlled clinical trial with 2 parallel arms 1:1 allocation ratio Registered	35 patients; Hybrid Hyrax = 20;(8F, 12 M) Mean age = 10.76yrs Conventional Hyrax = 15; (6F, 9 M) Mean age = 11.52 years	**Location:** Miniscrews in mandible distal to canines bilaterally **Protraction protocol:** Class III elastics full time started with 150 g/side in the first month and 250 g/side in the following period changed twice a day in the morning and at night **Expansion:** HH or CH 1/4 turn twice a day for 14 days, achieving 5.6 mm of expansion **Retention** Chin cup at night as active retention	- CBCT before Rx (T1) and after Rx (T2) -Surface superimposition and color map -Until a positive overjet is reached or a maximum of 12 months of treatment	-Shape analysis, volume and minimal axial area of airway were performed by 3D Slicer software via the SliceSALT project -Segmentations were performed in the ITK-SNAP software	The oropharynx volume and minimum axial area demonstrated a posttreatment increase in the HH group The oropharynx volume showed similar increases in both groups (MD: − 138.61; 95% CI: − 3078.01, 2800.80) Also, the minimum axial area showed similar increases in both groups (MD: 10.58; 95% CI: − 39.14, 60.30) Both groups showed similar upper airway increases after maxillary protraction
Beville et al., [36] 2012 Thesis	Prospective clinical study	30 patients 16 F, 14 M Mean age = 11.1 ± 1.1 yrs	**Location:** 4 miniplates placed on the right and left infra-zygomatic crest of the maxillary buttress and between the mandibular left and right lateral incisor and canine. Each of the miniplates was secured to bone with 2 or 3 screws **Protraction protocol:** Three weeks after surgery, Class III elastics were applied with an initial force of 150 g/side, and increased to a final force level of 250 g/side. The patients were instructed to wear the elastics 24 h/day. In some cases, a removable bite plate was used to eliminate occlusal interferences in the incisor area	CBCT 3D cephalometric analysis immediately after placement of miniplates (T1) Mean 1.1 years ± 1 month (T2)	Airway volume and area measurements were done using 3D Dolphin imaging software	Airway volume increased significantly an average of 1411.59 ± 2996.46 mm^3^ The area in the most constricted section of the airway increased slightly on average 13.11 ± 53.81 mm^2^, but this increase was not statistically different at T2 compared to T1
Nguyen et al., [35] 2015; Angle Orthod	Controlled clinical trial	10–14 yrs 28 patients (14F,14 M) Mean age = 11.9 yrs 28 controlled (16F 12 M) Mean age = 12.4 yrs	**Location:** Four miniplates placed, two in the infra-zygomatic crest of the maxillary buttress and two between the mandibular lateral incisors and canines **Protraction protocol:** Three weeks after surgery, the miniplates were loaded using Class III elastics applied at an initial force of 100 g on each side. The force was increased to 200 g after 1 month of traction and to 250 g after 3 months. The patients were asked to replace the elastics at least once a day and to wear them 24 h per day. In cases with increased overbite, a removable bite plate was inserted in the upper arch to eliminate occlusal interference	CBCT Before initial loading (T1) and after 1 yr (T2)	The airway volumes and minimum cross sectional area measurements were performed using Dolphin Imaging 11.7 3D software	From T1 to T2, airway volume from BAMP-treated subjects showed a statistically significant increase (1499.64 mm^3^) The area in the most constricted section of the airway (choke point) increased slightly (15.44 mm^2^) The airway volume of BAMP patients at T2 was 14,136.61 mm^3^, compared with 14,432.98 mm^3^ in untreated Class III subjects
Seo et al., [37] 2017 Maxillofacial Plastic and Reconstructive Surgery	Clinical trial	28 patients 8 M/ 20 F) were treated with a TBFM Mean age = 10.3 ± 1.4 yrs 24 patients (12 M /12F) were treated with an SAFM Mean age = 11.2 ± 1.1 yrs	**Location:** Two curvilinear miniplates with 1.5 mm thick **Protraction protocol:** **SAFM** Application of the facemask was begun 2 to 3 weeks after the surgery. Patients were asked to wear the face mask all day long and the protraction force of the elastics was adjusted to 400– 500 g on each side Vs **TBFM** with rapid palatal expansion therapy, in which the first maxillary premolars and first molar were banded with hooks on both sides Patients were instructed to activate the palatal expander one or two times a day until slight overexpansion was obtained. Patients in the TBFM group were asked to wear the face masks for at least 12 h a day. Approximately 400 g of elastic force was applied on each side	Lateral cephalometric before treatment (T1) and after treatment T2 Mean time TBFM = 14.3 months SAFM = 16.9 months	Linear measurement of superior, middle and inferior pharyngeal spaces; Areal measurements of superior, middle and inferior pharyngeal areas using V-ceph software	There were marked increases in upper airway dimensions in both groups following treatment, but the SAFM (skeletal anchored facemask) group had a significantly greater increase in airway dimensions than the TBFM (tooth borne facemask) group Also, the SAFM subgroups showed more improved airway measurements than the TBFM subgroups in both the superior and inferior pharyngeal airways

*HH* Hybrid Hyrax, *CI* Confidence Interval

*CH* Conventional Hyrax, *MD* Median Deviation

*CBCT* Cone Beam Computed Tomography, F Female, *M* Male

### Risk of bias in individual studies

**Fig. 2:**
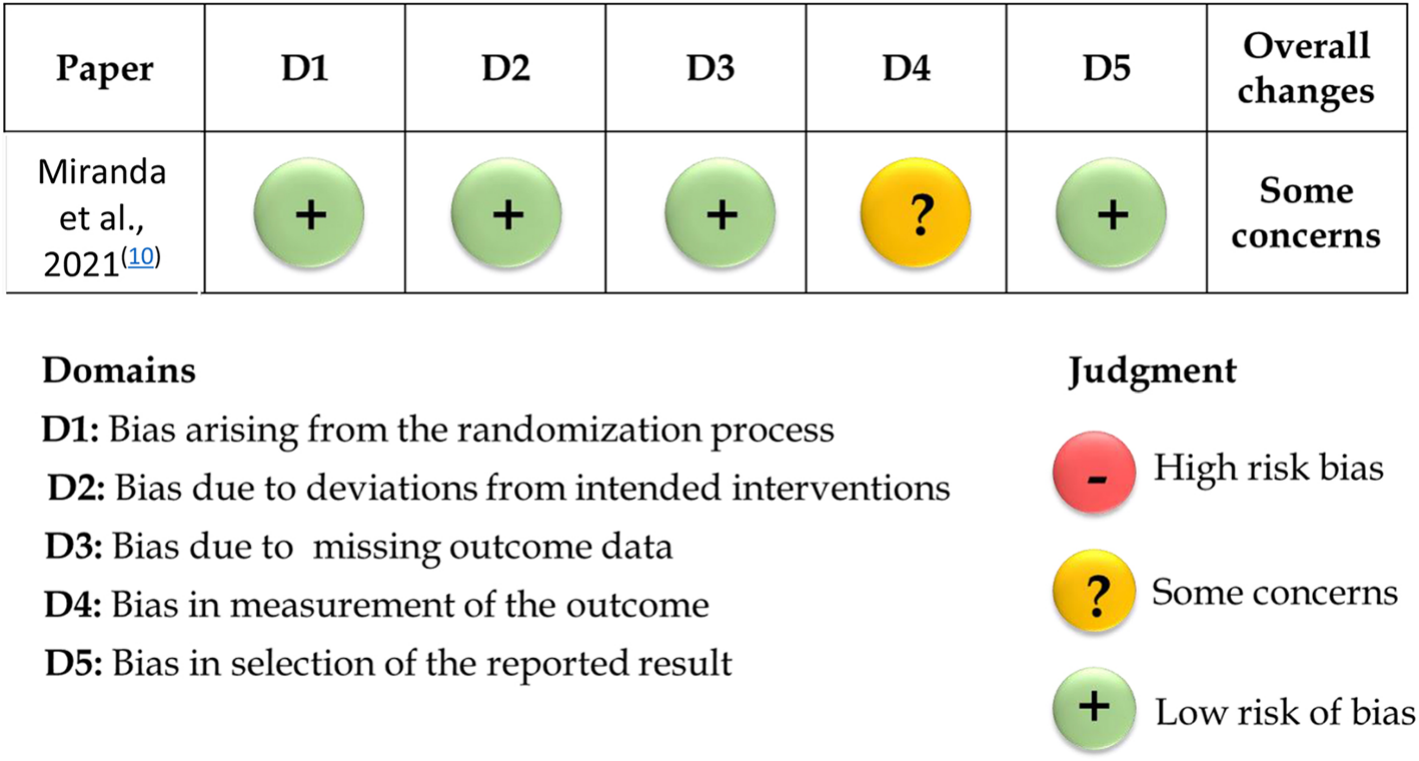
Summary of the risk of bias assessment according to Cochrane Collaboration’s RoB 2 tool for RCT

Assessment of bias risk was performed independently by two reviewers (S.A, B.A) using Cochrane Collaboration’s revised RoB 2 tool [36] for RCTs. Biases due to randomization; deviations from intended interventions; missing information; outcome assessment; selection of reported findings were all evaluated. If data was missing in any of the mentioned domains, the authors of the trial were reached out for clarification. Overall, the risk of bias of the study was considered “High” if any of the domains was judged as “High risk;” “Low” if all domains were judged as “Low risk,” and “Some concerns” if at least one domain was judged as “Some concerns”. Also, ROBINS-I tool [37] was utilized for evaluation of risk of bias for clinical trials. Bias due to confounding factors, selection bias, classification of intervention, deviations from intended interventions, missing data, and reporting bias were assessed. If data was missing in any domain, the authors of the study were contacted for clarification. Risk-of-bias could be judged as ‘Low’, ‘Moderate’, ‘Serious’ or ‘Critical’ risk of bias, with an additional option of ‘No information’. An overall risk of bias was considered “Serious” or “Critical” if any of the domains was judged as “Serious” or “Critical” respectively. It scored “Low” if all domains were judged as “Low risk”. Any arguments between the two reviewers were resolved by the discussion with the third reviewer (A.Z). (Table 3 and Fig. 2).

**Table 3:** Summary of ROBINS-I tool for non-randomized clinical trials

Paper	Confounding Bias	Selection Bias	Information bias	Reporting Bias	Overall bias
Beville et al., [36] 2012	Serious	Serious	Serious	Low risk	**Serious bias**
Ngyuen et al., [33] 2015	Low risk	Serious	Serious	Low risk	**Serious bias**
Seo et al., [37] 2017	Serious	Serious	Serious	Low risk	**Serious bias**

### Quality assessment in individual studies [38]

The modified Jadad score was employed to evaluate the quality of the eligible studies which would reflect the quality of the systematic review. The total quality of the paper was calculated with questions mentioned in Table 4. It was described as high quality if the paper scored >4, moderate quality if the paper scored 3–4 and low quality if the paper scored <3.

**Table 4:** Summary of modified Jadad score used for quality judgment

Questions			Miranda et al., 2021	Beville et al., 2012	Nguyen et al., 2015	Seo et al., 2017
Was the study described as randomized?			** + 1**	**0**	**0**	**0**
Yes	No					
+ 1	0					
Was the method of randomization appropriate?			** + 1**	**0**	**0**	**0**
Yes	Not described	No				
+ 1	0	-1				
Was the study described as blinded?			** + 0.5**	**0**	**0**	**0**
Double	Single	No				
+ 1	0.5	0				
Was method of blinding appropriate?			** + 1**	**0**	**0**	**0**
Yes	Not described	No				
+ 1	0	-1				
Was there description of withdrawals or dropouts?			** + 1**	** + 1**	**0**	**0**
Yes	No					
+ 1	0					
Was there a clear description of inclusion/ exclusion criteria?			** + 1**	**0**	** + 1**	** + 1**
Yes	No					
+ 1	0					
Was the method to assess adverse effects described?			** + 1**	**0**	**0**	**0**
Yes	No					
+ 1	0					
Was the method of statistical analysis described?			** + 1**	** + 1**	** + 1**	** + 1**
Yes	No					
+ 1	0					
Total			**7.5**	**2**	**2**	**2**
Quality description			**High quality**	**Low quality**	**Low quality**	**Low quality**

## Results

### Study selection

The procedure of study identification and screening is presented in Fig. 1. Electronic database search identified 779 articles. After omitting 399 duplicates, 380 records were screened by title and abstract to spot potentially eligible articles. Screening led to exclusion of 373 records and full texts of the resulting 7 articles were retrieved and analyzed carefully based on the eligibility criteria. Three articles were not eligible for the mentioned reasons: One study was a retrospective study [39] and two studies were pilot studies [40, 41]. Consequently, four studies [10, 33, 42, 43] were considered eligible for their inclusion in the current systematic review.

### Study characteristics

Characteristics of the included studies are summarized in Table 2. One study was a RCT with two parallel arms, [10] two clinical trials [42, 43] and one un-controlled clinical trial [33]. Two of the studies used rapid palatal expansion [10, 43] with the maxillary protraction, while in the other two studies, [33, 42] only the protraction protocol was done with different force magnitudes and protocols. Three of the studies used 3D CBCT as their method of airway change assessment to measure airway shape, volume and cross-sectional area. [10, 33, 42] On the other hand, only one study used 2D lateral cephalometric X-ray to measure linear and areal changes in airway dimensions. In the controlled clinical trials, the control groups were: conventional hyrax without facemask therapy in one study [10] and tooth-borne facemask therapy in the other study [43]. Skeletal anchorage devices varied from one study to the other. Miranda et al., [10] used two miniscrews in the lower jaw with class III elastics attached to the upper Hyrax. However, Beville et al. and Nguyen et al. [33, 42] used four miniplates in the upper and lower jaws with attached class III elastics. Finally, Seo et al. [43] used only two miniplates in the maxilla with facemask therapy.

### Risk of bias

The risk of bias for the eligible studies is summarized in Fig. 2 and Table 3. Applying RoB 2 tool in the RCT, the study was considered overall to show some concerns. This score was reached because domain 4 showed some concerns as there was no blinding for both the operator and the patients to the intervention. In the other three non-randomized clinical trials, ROBINS-I tool was used to evaluate their bias risk. The three studies were judged in total to show serious risk of bias, as there was at least one of the following domains: confounding, selection and information bias, showing serious risk of bias. Although, all of them showed low risk for the reporting bias domain.

### Quality assessment

When the modified Jadad score was used, only the RCT scored 7.5 revealing a high-quality study. On the other hand, the three non-randomized clinical trials scored 2 revealing low-quality studies, sharing a lack of randomization, blinding as well as reporting of the intervention's adverse effects (Table 4).

### Results of individual studies

All studies showed intra-operator reliability, where the Intraclass Correlation Coefficient values varied from 0.808 to 0.997 demonstrating good to excellent agreement of all measurements taken.

Miranda et al., [10] showed that both bone-borne maxillary protraction and the control group revealed a similar increase in the SNA and Wits appraisal. The oropharynx volume demonstrated similar rises in both groups (MD: − 138.61; 95% CI: − 3078.01, 2800.80). Additionally, the minimum axial area was shown to have similar increases in both groups (MD: 10.58; 95% CI: − 39.14, 60.30). However, no significant correlation was analyzed between the skeletal (SNA, SNB and Wits) and oropharyngeal effects (min Ax and OP volume) (*p* > 0.05 for all correlations tested). As for Beville et al. [42], the maxillary 3D linear measurements similarly showed a statistically significant (*p* < 0.001) increase in anterior–posterior dimensions of the maxillary arch. Regarding the airway dimensional changes, its volume raised significantly an average of 1411.59 ± 2996.46 mm^3^. Furthermore, the area in the most constricted section of the airway increased slightly on average 13.11 ± 53.81 mm^2^, but it was not statistically different at T2 when compared to T1.

Additionally, Nguyen et al., [33] demonstrated an increase in the SNA by 2.23°. The mean airway volume of the oropharynx demonstrated a statistically significant increase from T1 (12,636.89 mm^3^) to T2 (14,136.61 mm^3^). The midsagittal area revealed a statistically significant increase, and the minimum cross-sectional area increased slightly from 148.21mm^2^ to 163.65mm^2^, although this was not statistically significant. There was no statistical difference between the treated and the untreated control groups in airway volume, midsagittal area, and minimum cross-sectional area respectively (BAMP 14,432.98 mm^3^, 674.36 mm^2^, and 174.56 mm^2^; control 14,560.33 mm^3^, 643.67 mm^2^, and 170.94 mm^2^).

Finally, Seo et al., [43] reported a mean value of 2.74 mm maxillary advancement in the TBFM group. On the other hand, the mean value of maxillary protraction in the SAFM group was 3.63 mm, which was greater than the other group, but not in a significant manner (*p* > 0.05). An increase in the pharyngeal airway measurements was noticed in TBFM (tooth borne facemask) and SAFM (skeletal anchored facemask) groups. For the linear measurements, SPPS increased in the two groups after treatment but with no statistically significant difference (*p*>0.05). On the contrary, a significant increase was reported in the MPS (middle pharyngeal space) and IPS (inferior pharyngeal space) in the SAFM group when compared to the TBFM group (MPS *p*˂0.05, IPS *p*˂ 0.01). Regarding the areal measurements, the SPPA (superior pharyngeal area) improved significantly in SAFM group in comparison to TBFM group (*p* ˂ 0.01), whereas MPA (middle pharyngeal area) increased in both groups but without a remarkable difference between them. Noticeably, IPA (inferior pharyngeal area) didn’t increase significantly after treatment.

## Discussion

### Summary of evidence

The objective of the present systematic review was to appraise evidence from randomized and nonrandomized prospective clinical trials on airway changes following the utilization of bone-anchored maxillary protraction devices. Previously, systematic reviews and meta-analyses either compared the treatment effects of bone-anchored maxillary protraction skeletally, dentally and on the soft tissues or compared airway changes between different tooth borne protraction and expansion appliances [25]. However, up to our knowledge, the current systematic review is the first to compare the effects of bone anchored maxillary protraction on the oropharyngeal airway dimensional changes. The principal findings were that the more the maxillary protraction, the more the airway changes occurring but not necessarily in a significant manner.

### Study designs and control groups

Searching through the literature, after applying the eligibility criteria, yielded four studies: including one RCT, [10] two controlled clinical trials [33, 43] and one uncontrolled clinical trial [42]. The control groups were either positive controls: using conventional expansion [10] and tooth-borne face mask therapy, [43] or negative controls: untreated group [33].

### Mean age range

The mean age range in all studies was between 10.3 and 11.9 years. Taylor et al., [44] reported that most posterior pharyngeal airway growth occurs in two different growth spurts from 6 to 9 years and from 12 to 15 years. Therefore, development in the airway dimensions from 9- 12 years is considered to be negligible. Upon contrasting and comparing the age range used in the included studies, it was found that Beville et al., [42] and Seo et al., [43] used mean age from 10 – 11 years which was between the two growth spurts in order to omit the growth as a confounding variable in analyzing airway changes results. On the other hand, Miranda et al., [10] and Nguyen et al., [33] used an age range from 11.5 to 11.9 years before treatment and therefore the post-treatment airway assessment was coinciding with the second growth spurt. Hence, their results of airway increase due to bone-anchored maxillary protraction should be taken with caution as it wasn’t purely from the maxillary protraction itself.

### Expansion in conjunction with the bone anchored maxillary protraction

It was only done in the RCT conducted by Miranda et al., [10] Therefore, the rise in the airway dimensions from this study can’t be exclusively attributed to the maxillary protraction leading to bias.

### 2D versus 3D method of airway assessment

Conventionally, 2D lateral cephalometric X-rays were used to assess linear and areal airway changes as used in Seo et al.,[43] study. However this method has its well-known limitations [30, 45, 46] including distortion and position errors, inability to measure 3D volumes and inability to reflect the true anatomical airway structure. Seo et al., [43] demonstrated a remarkable increase in some airway measurements, without significant increase in others. This is in agreement with Baccetti et al., [24, 28] and Tuncer et al., [47] who also used 2D lateral cephalometric X-ray and found no significant change in airways following maxillary protraction. With the advancement of technology, the CBCT added the missed third dimension to the 2D image. Additionally, the CBCT is considered reliable for measuring airway volumes, areas and shapes. Moreover, it has no error of magnification and parallax occurring in the 2D image. In the other three included studies in the current systematic review, CBCT was used to evaluate the volumetric, areal and shape changes, demonstrating an increase in the previously mentioned airway dimensions. This was in agreement with Kaygisiz [48] who revealed short and long-term improvements in nasopharyngeal and upper airway changes that were retained for four years post-retention.

Variable conditions during image acquisition could have an effect on the recorded airway dimensions including inspiration and expiration, supine versus upright position, neck flexure and scan time. Hence, these factors should be controlled during radiographic image acquisition, as well as the implementation of other methods of assessment like airflow monitors that could be used for evaluating respiratory efficiency.

### Observational period

For all the studies included, [10, 33, 42, 43] the longest observational period was 17 months which was not enough to evaluate the long-term improvement of the airway changes.

### Different bone anchored protraction devices and protraction protocols

Eligible studies included in the presented systematic review presented different bone anchored protraction devices with different protocols [10, 33, 42]. For example, Miranda et al., [10] used Hyrax expander with class III elastics to two mini-screws placed in the lower jaw. The elastics were changed twice daily. While in Beville and Nguyen et al., [10, 42] studies, four miniplates were placed in the upper and lower jaws with which class III elastics were attached and changed once daily. Differently, Seo et al., [43] used two miniplates in the maxilla attached to an extra oral facemask with elastics changed once daily.

Although different designs for bone-anchored protraction were used in different studies, they all showed an increase in the airway dimensions, which was in agreement with previously reported studies by Hiyama and Kaygisiz et al., [48, 49] Moreover, our positive findings for airway dimensions are in accordance with the results of a meta-analysis conducted to investigate the changes in airway dimensions after tooth borne rapid maxillary expansion (RME) and facemask (FM) protraction. The meta-analysis demonstrated that statically significant changes in upper airway and nasal passage airway were observed in the intervention groups as compared to the control groups based on the nine included studies in their assessment [25]. Nevertheless, this was opposite to the results reported by Bacetti et al., Tuncer et al., Mucedero et al., and Pamporakis et al., [24, 28, 47, 50] who showed no significant difference in airway dimensions between treated groups using either facemask or chin cup compared to the control groups. The conflict in results between these studies and the eligible studies could be explained by the use of bone anchored devices in this review.

There are many controversies observed when analyzing the magnitude of the protraction forces used in different studies. Most of them [10, 33, 42] applied 150 gms/ side initially that was increased later to reach 250 gms/ side. However, Seo et al., [43] used 400 -500 gms / side. Nevertheless, none of the previously mentioned studies justified the magnitude of the protraction force applied.

### OSA and maxillary protraction

The change in airway dimension is not necessarily correlated with the physical function. This means that even significant increases in airway dimensions don’t necessarily imply a clinical improvement in airway problems. There are uncertainties about the airway increase after maxillary protraction. This could be explained by the protraction force that induces forward growth of the maxilla especially posterior nasal spine leading to the forward displacement of the soft palate with consequent rise in the airway dimensions [49]. Another explanation could be the forward positioning of the tongue which is modified by the facemask increasing the volume of the oral cavity [50]. Recent studies [49, 51–53] showed a strong correlation between minimal cross sectional area of oropharynx and obstructive sleep apnea. They found that maxillary protraction was closely associated with increase in respiratory function and alleviation of respiratory discomfort. However, up till now there is shortage in the number of studies reporting the resulting effects of maxillary protraction on the outcomes of OSA using specific diagnostic tools as polysomnography.

### Limitations

Lack of untreated control groups to be compared with the treated groups is considered a limitation of the present review. However, this was justified by the ethical considerations regarding radiation exposure to untreated groups and leaving skeletal class III patients untreated. Moreover, there were several confounding variables that might lead to bias including whether expansion was performed in conjunction with protraction or not and 2D versus 3D assessment methods.

There were limitations in the design of some of the bone anchored devices like mentioned in Seo et al., [43] in which screw loosening was reported because the plates and screws were not principally fabricated for skeletal anchorage and need to be altered preoperatively. In addition to the presence of the hook at the last hole having inside threads for locking which had sharp edges that resulted in elastics break. Therefore, the plate and screw system need to be optimized.

### Recommendations for future research

Research is needed in the future to compare different bone anchored maxillary protraction devices with different protocols to each other excluding growth factor and also excluding expansion before or during protraction to avoid any confounding factors leading to bias. Additionally, more specific ways of OSA assessment should be used to assess the maxillary protraction effect on OSA outcomes solely. Last but not least, modification in design of some bone anchored devices to optimize the load applied from the protraction devices is needed.

## Conclusion

Based on the available evidence, all the bone-anchored maxillary protraction devices used in the eligible studies in the present systematic review resulted in an improvement in the airway dimensions. However, due to the few numbers of studies available and the guarded evidence due to the low quality of evidence of three out of four included articles, there is no strong evidence to support a significant increase in the airway dimensions following bone-anchored maxillary protraction. There is a need for more randomized controlled clinical trials with similar bone-anchored protraction devices and similar assessment methods for more valid comparisons, excluding the growth effect on airway dimensional changes.

## Data Availability

All data generated or analysed during this study are included in this published article in the form of tables and figures.
